# The CareWell-primary care program: design of a cluster controlled trial and process evaluation of a complex intervention targeting community-dwelling frail elderly

**DOI:** 10.1186/1471-2296-13-115

**Published:** 2012-12-05

**Authors:** Franca GH Ruikes, Antoinette RM Meys, Gijs van de Wetering, Reinier P Akkermans, Betsie GI van Gaal, Sytse U Zuidema, Henk J Schers, Theo van Achterberg, Raymond TCM Koopmans

**Affiliations:** 1 Department of Primary and Community Care, Radboud University Nijmegen Medical Centre, P.O. Box 9101, Nijmegen, HB 6500, the Netherlands; 2 Department of Primary and Community Care / Scientific Institute for Quality of Healthcare, Radboud University Nijmegen Medical Centre, P.O. Box 9101, Nijmegen, HB, 6500, the Netherlands; 3 Scientific Institute for Quality of Healthcare, Radboud University Nijmegen Medical Centre, PO Box 9101, Nijmegen, HB, 6500, the Netherlands; 4 Department of General Practice, University of Groningen, University Medical Centre Groningen, P.O. Box 196, Groningen, AD, 9700, the Netherlands

**Keywords:** Frail elderly, Complex intervention, Integrated care, Functional status, Cost-effectiveness, Implementation, Process evaluation, Primary care

## Abstract

**Background:**

With increasing age and longevity, the rising number of frail elders with complex and numerous health-related needs demands a coordinated health care delivery system integrating cure, care and welfare. Studies on the effectiveness of such comprehensive chronic care models targeting frail elders show inconclusive results. The CareWell-primary care program is a complex intervention targeting community-dwelling frail elderly people, that aims to prevent functional decline, improve quality of life, and reduce or postpone hospital and nursing home admissions of community dwelling frail elderly.

**Methods/design:**

The CareWell-primary care study includes a (cost-) effectiveness study and a comprehensive process evaluation. In a one-year pragmatic, cluster controlled trial, six general practices are non-randomly recruited to adopt the CareWell-primary care program and six control practices will deliver ‘care as usual’. Each practice includes a random sample of fifty frail elders aged 70 years or above in the cost-effectiveness study. A sample of patients and informal caregivers and all health care professionals participating in the CareWell-primary care program are included in the process evaluation. In the cost-effectiveness study, the primary outcome is the level of functional abilities as measured with the Katz-15 index. Hierarchical mixed-effects regression models / multilevel modeling approach will be used, since the study participants are nested within the general practices. Furthermore, incremental cost-effectiveness ratios will be calculated as costs per QALY gained and as costs weighed against functional abilities. In the process evaluation, mixed methods will be used to provide insight in the implementation degree of the program, patients’ and professionals’ approval of the program, and the barriers and facilitators to implementation.

**Discussion:**

The CareWell-primary care study will provide new insights into the (cost-) effectiveness, feasibility, and barriers and facilitators for implementation of this complex intervention in primary care.

**Trial registration:**

The CareWell-primary care study is registered in the ClinicalTrials.gov Protocol Registration System: NCT01499797

## Background

Worldwide, an increase in life-expectancy and ageing of the baby boom generation is leading to a vastly expanding population of elders. In the Netherlands, the number of people aged 65 years or above will increase from 2.4 million in 2010 to 4.6 million in 2040. Furthermore, life expectancy in the Netherlands will increase from 78.8 years to 84.5 years for males and 82.7 years to 87.4 years for females in the time span [1].

Advancing age often implies an increase in the incidence of chronic diseases and multi morbidity with subsequent functional decline and social impairments, e.g. the loss of social support, financial limitations, and the lack of appropriate housing [2,3]. The current system of health care delivery for community-dwelling frail elder people, with these numerous and complex health-related needs, is insufficient due to fragmentation and a lack of coordination and information exchange between health care professionals. Furthermore, sophisticated health information technologies that facilitate the essential processes of chronic care are not widely in use [4,5]. Moreover, less urgent needs to optimally manage chronic illness and care for health related social and welfare problems are overshadowed by acute symptoms and concerns [6,7]. Last, payment for and provision of medical and nursing care and social services are separated rather than integrated, and payment policies do not support supplemental services needed in providing chronic care [4,5].

Frail elderly people are believed to benefit greatly from a coordinated chronic health care delivery system that integrates health and social care [8]. A variety of models have been developed and tested over the last twenty-five years [9,10]. This gave rise to an emerging vision of an optimal chronic care model in which health care organizations give priority to chronic care, health care providers are linked to community resources, chronic care management is separated from the acute care, elders receive self-management support, and evidence-based guidelines and clinical information systems are available to facilitate chronic care management [6,7].

Few studies on such comprehensive chronic care models targeting frail elder persons have been conducted. Positive effects on functional performance [11], on self-reported quality of health care [12], and on informal caregiver satisfaction [13] are suggested, although overall (review) findings are inconsistent [14,15]. Furthermore, previous studies have shown some cost-saving implications through a postponement or reduction in residential or nursing home admissions, hospital admissions and emergency department visits [11,13,16-18].

The CareWell-primary care program is a complex intervention integrating cure, care and welfare, that aims to prevent functional decline, improve quality of life and reduce or postpone hospital admissions and nursing home admissions in community-dwelling frail elderly. The program is based on existing chronic care models and is adapted to the Dutch health care system. It is designed as part of the National Care for the Elderly Program, which is launched in 2008 by the Netherlands Organization for Health Research and Development (ZonMW), in cooperation with the Nijmegen Network for the Care and Welfare of Elderly People [4]. In developing the program, both health care professionals and a panel representing frail elderly and their informal caregivers were closely involved.

Complex interventions comprise multiple components that are interrelated or interdependent and therefore can be difficult to develop, document, evaluate and reproduce [19]. To create a better understanding of how and why a complex intervention works, and to gain insight into costs and benefits, the framework for development and evaluation of complex interventions as published by the UK Medical Research Council is widely used [20]. This framework emphasizes the value of including a process evaluation and an economic evaluation alongside the outcome evaluation. It provided the theoretical background for the design of our study. By gaining process information, we aim to detect gaps in implementation that might be responsible for the effectiveness of the program. Furthermore, we will explore why some general practices are more successful than others in improving the quality of care for their frail elderly patients [21].

This paper presents the elements of the CareWell-primary care program as well as the design of both the CareWell-primary care (cost-) effectiveness study and process evaluation.

## Methods/design

### Study design and setting

The CareWell-primary care study has a pragmatic, cluster controlled design [22]. It will be conducted in 12 general practices in (the municipality of) Nijmegen, the Eastern region of the Netherlands.

### Study population

#### Recruitment of general practices

General practitioners (GPs) are recruited to participate in the CareWell-primary care program through an invitational letter and a subsequent telephone call from one of the principal investigators. They are fully informed on the EasyCare Two-step Older persons Screening instrument (EasyCare-TOS) [23], used to identify the frail elderly study participants, and on the elements of the program. GPs with a minimum of 300 patients aged 70 years or above in their practice population, a solid motivation to implement the program, and the organizational facilities required for implementation are eligible to participate in the intervention arm. After their informed consent, six GPs will be non-randomly assigned to the intervention arm.

A second group of GPs is similarly recruited to participate in the control group. These GPs receive information on the EasyCare-TOS, but no information on the CareWell-primary care program in order to prevent contamination bias. Furthermore, they are explicitly instructed to deliver ‘care as usual’, and not to start new collaborations with community nurses, elderly care physicians or gerontological social workers. No restrictions on existing collaborations are imposed. However, no multidisciplinary team collaborations comparable to those in the CareWell-primary care program are regularly available in usual care. Six GPs consenting to participation are non-randomly assigned to the control arm.

#### Study participants in the cost-effectiveness study

In each general practice, a random sample of fifty frail elders aged 70 years or above will be included in alphabetical order with the use of the EasyCare-TOS screening instrument [23]. In step 1 of the EasyCare-TOS, the GP rapidly subdivides ‘not-frail’ from ‘(possibly-) frail’ elders by using prior, tacit knowledge. (Possible) Frail elders proceed to step 2, in which a trained community nurse or research assistant conducts a comprehensive geriatric assessment during a home-visit. The EasyCare-TOS step 2 questionnaire is shown in Additional file 1. In both study arms 300 frail elders will be included. Excluded from participation are (1) elders living in a residential or nursing home, (2) critically or terminally ill elders, (3) elders who are already enrolled in a case-management program, comparable to the CareWell-primary care program.

#### Informed consent

Eligible elders are asked for their willingness to participate in step 2 of the EasyCare-TOS and, in the intervention arm, in the CareWell-primary care program. Interested elders subsequently receive a written letter containing information on the EasyCare-TOS and, in the intervention group, the CareWell-primary care program. Finally, written informed consent is collected during the home-visits.

#### Study participants in the process evaluation

Next to a sample of patients and informal caregivers, all health care professionals participating in the CareWell-primary care program are included in the process evaluation; the GP’s, community nurses, gerontological social workers and elderly care physicians.

### Ethical considerations

The study has been reviewed by the local accredited medical review ethics committee: CMO region Arnhem-Nijmegen, registration number 2010/403. They concluded that formal ethical approval is not required, since the study does not involve research as covered by the Medical Research Involving Human Subjects Act. The study is registered in the ClinicalTrials.gov Protocol Registration System: NCT01499797.

### The intervention: the CareWell-primary care program

**Figure 1:**
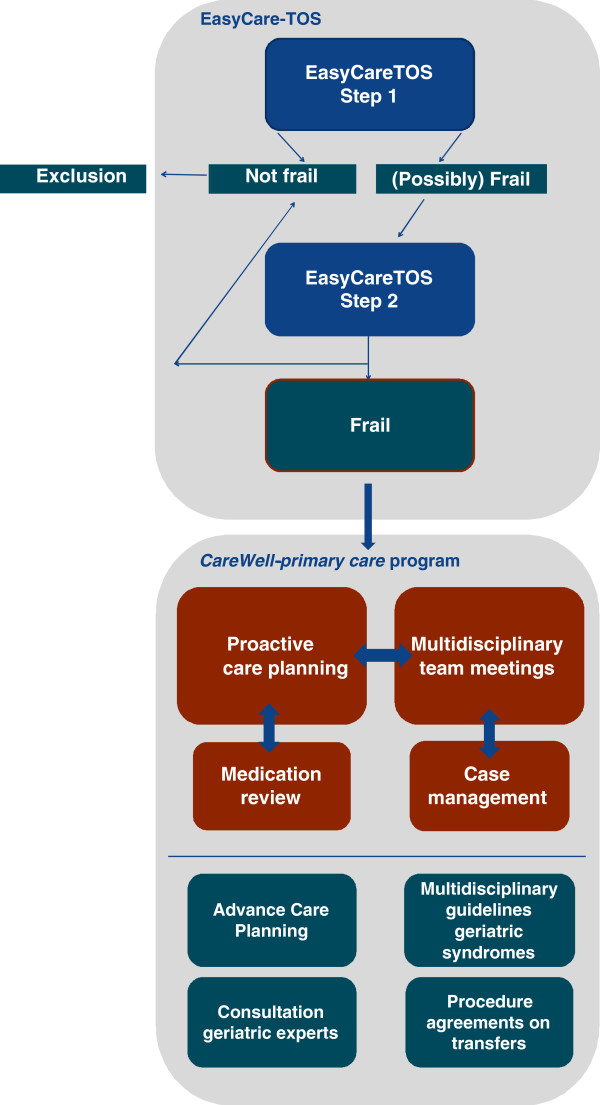
Schematic representation of the EasyCare-TOS and the elements of the CareWell-primary care program.

During a twelve-month intervention-period, the frail elders in the intervention group receive care according to the CareWell-primary care program. Figure 1 shows a schematic representation of the EasyCare-TOS and the elements of the CareWell-primary care program.

The program consists of four key elements: (1) multidisciplinary team work, (2) proactive care planning, (3) case management, and (4) medication reviews. Each general practice will assemble one or two multidisciplinary teams, consisting of the GP, the community nurse, an elderly care physician and a gerontological social worker. These team members closely collaborate to ensure integration of cure, care and welfare. Face-to-face multidisciplinary team meetings will be held at least twice a year for each frail elder, in which care plans will be reviewed and adapted. In addition, the team members will be able to virtually communicate at all times within a secured web based health and welfare information portal (ZWIP) [24]. This portal combines a shared Electronic Health Record with a communication tool for primary care professionals, which is accessible to all involved caregivers through a secured login procedure.

A proactive integrated care plan is formulated for each participant on enrollment in the program. These care plans will be based on the individual patients’ health-related goals and needs on the domains of cure, care and welfare as obtained with the EasyCare-TOS. The care plans will be stored in the ZWIP.

All elders will be assigned a case manager. This will be either the community nurse or the gerontological social worker, depending on the nature of the participants’ health-related needs. The case manager will be responsible for the organization of the multidisciplinary team meetings and for the coordination and monitoring of the proactive care process according to the care plan, as directed by the primary care physician. Moreover, the case manager will provide participant-support in goal setting and self-management by means of home-visits and telephone contacts.

The GP, community nurse and pharmacist will conduct a yearly medication review for those elders using five or more drugs for chronic use. Agreements on discontinuing inappropriate or unnecessary medications and starting medications in case of under-treatment will be incorporated in the care plan, thus ensuring appropriate drug treatment [25].

In addition to these four key elements, four supporting elements facilitate the care delivery according to the program. First, we developed multidisciplinary practice guidelines on the medical treatment and nursing and social care of eight common geriatric syndromes: depression, dementia, chronic pain, falls, urinary incontinence, malnutrition, and vision and hearing impairment. These guidelines are presented as a job aid in the ZWIP. Second, a practice guideline concerning advance care planning is developed and presented in the ZWIP to promote a proactive dialog between frail elders and their GPs on wishes and expectations regarding medical treatment and end-of-life decisions. Third, procedure agreements regarding easy-access consultation of a geriatrician or a geriatric psychiatrist are constructed. Last, procedure agreements on hospitalization and discharge are made to facilitate the integration of primary- and in-hospital care, thus improving the interdisciplinary continuum of care.

### Tailored implementation strategies

At baseline, health care professionals in the intervention group are asked for their perceived barriers in the current practice of elderly care as well as for their expectations of the CareWell-primary care program by means of a structured questionnaire. This questionnaire is based on the baseline questionnaire developed in the Dutch Easy Care study [26] and is pilot tested with peer group professionals. The information thus collected will be used to tailor the implementation strategies and activities, in order to facilitate optimal implementation of the CareWell-primary care program. A combination of implementation strategies and activities targeting both health care professionals and organizations will be used, addressing a variety of barriers for change [27]: (1) different types of education (tailor-made meetings, coaching on the job, a helpdesk, and expert meetings) to overcome gaps in knowledge, attitude and skills needed to conduct the program, (2) persuasive communication and social influencing by means of large group meetings, in order to enhance both motivation and endurance for participation, (3) provision of additional information through a website, newsletters and written instructions, (4) providing feedback and advice to the participating professionals, and (5) financial reimbursement for all health care professionals and organizations to cover the extra efforts required by the program, to facilitate participation in the intervention. These implementation activities will start nine months before the actual start of the program and will be continued throughout the program.

### Cost-effectiveness study

#### Outcome measures and data collection

The primary outcome is the change in the level of functional performance in ADL between baseline and follow-up at twelve months, as measured with the Katz-15 index [28].

Secondary outcomes are:

1. Quality of life, as measured with RAND-36 [29] and EQ-5D [30]

2. Psychological and social functioning, as measured with a subscale of the RAND-36 [29]

3. Number of residential home, nursing home and hospital admissions

4. Mortality

Participants’ data are collected at baseline and at follow-up at twelve months with the EasyCare-TOS, in which baseline characteristics, the Katz-15 index, RAND-36, EQ-5D, and data on health service utilization and mortality are embedded.

Caregiver burden is measured with the Carer-Qol [31], which is embedded in a structured caregiver questionnaire, to be filled in by the main informal caregiver.

Last, regular health care costs and costs of the CareWell-primary care program are collected with the EasyCare-TOS and through external sources, as shown in Table 1.

**Table 1:** Overview of sources used to obtain regular health care costs and costs of the CareWell-primary care program

**Costs**	**EasyCareTOS**	**External source**
**Regular health care**		
regular GP contacts	X	electronic health record
out-of-office hours GP contacts	X	-
home care	X	home care organization
domestic care	X	municipality
medication	X	electronic health record
residential home admissions	X	-
nursing home admissions	X	-
day care in residential home	X	-
day care in nursing home	X	-
hospital admissions	X	-
physiotherapist	X	-
assistive devices	X	-
**CareWell-primary care program**		
time needed for proactive care planning / case management / multidisciplinary deliberation medication review	-	time registrations by health care professionals

#### Sample size calculation

The change in functional status between baseline and follow-up will be measured as a change in the sum-score on the Katz-15 index between baseline and 12 months [28]. Although the Katz-15 index scores may be skewed, we expect these sum-score differences to have a normal distribution. For financial and logistic reasons, including 6 clusters in each study-arm is thought to be feasible. Each general practice is instructed to include 50 frail elderly. Based on the assumptions that 15% of eligible elders will decline informed consent and 20% will be lost to follow-up within the intervention-period of twelve months, the expected cluster size is 35. Using a two-sided alpha of 0,05, a power of 80%, an assumed between-clusters ICC of 0,01 [32], and a minimum cluster size of 35 with 2×6 clusters, we will be able to detect an effect size of >0,32, which is sufficient to detect even small differences [33,34].

#### Statistical analysis

The primary analysis will be performed adhering to the intention-to-treat principle. Descriptive statistics will be used to summarize characteristics of both participants and practices. Since the study has a hierarchical structure in which participants are nested within general practices, we will use hierarchical mixed-effects regression models / multilevel modeling approach to evaluate differences between the intervention and the control group in change in functional abilities between baseline and follow-up as measured with the Katz-15 index and all secondary outcomes. We will correct for the relevant covariates. Furthermore, the effect of the intervention on mortality, and on the time to hospital and nursing home admissions will be analyzed using survival analysis (Kaplan-Meijer curves) and Cox proportional hazard regression models. An additional sensitivity analysis will be conducted on a per-protocol analysis set, and on a subset of general practices in which the intervention is optimally implemented. Interim analyses will not be conducted. Statistical analyses will be performed using SPSS version 20.

#### Economic evaluation

The economic evaluation will be conducted from a societal perspective. All relevant direct and indirect costs per participant will be determined by considering costs of the CareWell-primary care program, for the intervention group, and regular health care costs, for both the intervention and the control group. The costs of the CareWell-primary care program will be calculated from the registrations of health care professionals of the time spent on the elements of the program. Regular health care costs will be collected with EasyCare-TOS and external sources, as shown in Table 1.

Unit resource prices are based on guideline prices according to the Dutch Insurance Board [35]. Real costs prices will be determined when unit resource prices are not available. Societal costs are quantified by calculating productivity losses for informal caregivers who perform paid labor during the study period using the friction cost method [36]. Data on productivity losses will be obtained using the structured caregiver questionnaires.

The incremental cost-effectiveness ratio (ICER) will be expressed as costs per quality-adjusted life years (QALYs) gained, as measured with the Euroqol-5D [30]. From these EQ-5D scores, utilities will be derived using the trapezium rule and the Dutch algorithm after which QALYs will be calculated [37].

Next to this, the ICER will be expressed as the difference in total mean costs weighed against the difference in the sum-scores between baseline and follow-up on functional performance, as measured with the Katz-15 index [28].

Both ICERs are subjected to bootstrap analysis and will be presented in cost-effectiveness planes. Deterministic uncertainty will be explored on a range of extremes of parameters potentially influencing the ICERs, i.e. sensitivity analyses. Furthermore, stochastic uncertainty surrounding the ICERs will be presented using a cost-effectiveness acceptability curve.

### Process evaluation

#### Outcome measures and data collection

Our extensive process evaluation aims to answer the questions: To what extent is the CareWell-primary care program implemented? How do patients, informal caregivers and professionals engage with and approve of the program? What are the barriers and facilitators to implementation?

The process evaluation is based on the steps for developing a process-evaluation plan provided by Saunders et al. [38], adapted from Steckler and Linnan [39]. This framework describes the following components: context, recruitment, reach, dose delivered, dose received, and fidelity. In this process evaluation, we will use mixed methods, i.e. both qualitative and quantitative methods. Table 2 shows the methods and instruments used in the process evaluation (1–11). Implementation fidelity and dose delivered, referring to the completeness of the delivery of the program, will be measured by (1) file analysis, (2) structured observation, and (3) analysis of time registration. In the examination of patients’ files in the ZWIP, the implementation rate of the four key elements of the program will be noted. Scores will be compared between general practices. In addition, two independent assessors will observe the practice teams during a multidisciplinary team meeting. A structured checklist that is based on the working instructions of the program will provide insights in elements concerning the organizational aspects of the meeting, the preparation of the participants, and the process of goal setting, action planning, monitoring and evaluation. Scores on the observed elements will be analyzed per general practice and per professional discipline. Inter-assessor reliability will be established by calculating Cohen’s Kappa. Time registrations will be analyzed to evaluate variation in the course of time of the intervention period, variation between individual health care professionals within the same discipline, the distribution off spent time over the different categories of activities. Data on the approval of patients and informal caregivers concerning the program and its key elements, will be gathered through (4–5) structured questionnaires and semi-structured interviews with patients and informal caregivers. These questionnaires will be based on both the Dutch translation of PACIC [40,41], and the CQ index [42], and adapted to our program. The results of the questionnaires will be compared to the key elements of the CareWell primary care program. Following this, semi-structured interviews with patients and informal caregivers will provide deeper insight in their experiences and relevant context factors. Furthermore, information on health care professionals’ views on the completeness of, exposure to, and satisfaction with the implementation activities will be collected and related to context variables, through (6–7) the registration and evaluation of educational meetings, (8–9) the registration of site visits and e-mail contacts between investigators and general practices, and (10–11) both structured questionnaires, reflecting on the professionals’ baseline expectations of the program, and focus group meetings. The interviews with patients and informal caregivers will be audio taped and reported by an independent observer. The focus group meetings will be audio taped, observed and reported.

**Table 2:** Methods and instruments used in the process evaluation

***Research question (outcome)***	***Components***	***Methods and instruments***
***1. Level of implementation***	***Fidelity Dose delivered (completeness)***	1. File analysis on web based patients files: presence of actual care plan per patient, domains concerned (somatic, functional, community participation, psychological, communication), planned and performed evaluations, team meeting reports, content of and professionals concerned in digital communication, registration of medication reviews.
		2. Observation of team meetings by means of a structured checklist: attendance, preparation, goal setting, evaluation appointments, monitoring results.
		3. Time registration form for professionals, collected by e-mail.
***2. Engagement and approval of patients and informal caregivers***	***Dose received (exposure)***	4. Structured questionnaire verbally collected from a sample of patients and informal caregivers. Items: engagement of patient in care plan, given choices and priorities, support, encouragement, cooperation between case manager and primary care physician.
	***Dose received (satisfaction)***	
		5. Semi-structured interviews with a sample of patients and informal caregivers on the same items to deepen the outcomes of the structured questionnaires.
***2. Engagement and approval of professionals***	***Dose delivered (completeness)***	6. Registration of attendance of educational meetings.
***3. Barriers and facilitators to Implementation***	***Dose received (exposure)***	7. Structured evaluation form for educational meetings.
	***Dose received (satisfaction) Context***	8. Registration of site visits: frequency, duration and content.
		9. File analysis on e-mail correspondence between program facilitator and teams.
		10. Structured questionnaire, electronically collected from all participating professionals.
		Items: relevance and feasibility of the program, extent to which the program was performed, interactions with staff and investigators, factors at individual, organizational and environmental levels that may have influenced the implementation of the program.
		11. Focus groups with a sample of participating professionals to deepen the outcomes of the structured questionnaires.

#### Statistical analysis

Descriptive statistics will be used in the analysis of the quantitative data coming from the patient’s web based files, team meetings observations, time registration results, patient’s questionnaires, attendance and approval of educational meetings, registration of site visits and e-mail correspondence, and structured questionnaires for health care professionals. Next, qualitative analysis will be performed on the interview data with patients and informal caregivers, and on the results of the focus group meetings for health care professionals, according to the method of open and axial coding [43], and with support of Atlas-TI software for qualitative analysis.

## Discussion

The CareWell-primary care program is a unique program for community dwelling frail elderly for several reasons. First, it targets frail elderly aged 70 years and above with and without care-complexity. Second, it focuses on extensive collaboration between health care professionals in primary care for elders; not only GPs and community nurses are involved, but also elderly care physicians to contribute their specific geriatric expertise, and, to be stressed, gerontological social workers in order to achieve comprehensive integration of welfare issues in the care for the elderly, that commonly has a focus on medical aspects of care. Last, it uses a secured, easily accessible web-based health and information portal (ZWIP) [24], in which care plans and guidelines are stored in patients’ files, that facilitate interdisciplinary consultation and communication complementary to the ‘live’ multidisciplinary meetings.

The CareWell-primary care program collects a minimum data set of baseline characteristics and outcome measures. Within the Dutch National Care for the Elderly Program [4], these data will be openly shared in order to serve public interest, advance knowledge and, last but not least, to be able to compare outcomes of the different research projects [44,45].

### Strengths and limitations

Since the CareWell-primary care program demands a thorough shift from reactive, acute-disease management to proactive, integrated, chronic care management that involves multiple health care professionals, the implementation of the program demands strongly motivated professionals working in adequately equipped practice settings. Interested GPs are therefore fully informed on the elements of the program to assure their motivation to participate, and their eligibility. Following their informed consent, they are non-randomly assigned to the intervention arm. Study participants are clustered within the general practices of these GPs. As a result of this recruitment strategy, the participating GPs may be atypically well motivated or resourced, influencing the external validity. Recognizing this, we will use the knowledge of facilitators and barriers to achieve further implementation of the CareWell-primary care program to other regions in the Netherlands.

In recruiting both the intervention and the control practices, no restrictions are made in baseline characteristics of the GPs, such as working experience, nor in the practice settings, such as existing collaborations between professionals and caregivers in primary care. Moreover, the conduction of the CareWell-primary care program is not subjected to standardization, other than the minimum requirements of twice-yearly multidisciplinary meetings, appointing a case manager to each participant and conducting yearly medication reviews. The subsequent heterogeneity in practice settings and in the delivery of the program will further enhance the generalizibility of our study.

The control group in our study receives ‘care as usual’. An important question to be answered is: “How usual is usual care in the control group?” [46]. Since the participants in the control arm are included with the EasyCare-TOS, it is very well possible this will change their health-seeking behavior. Also, the professionals in the control group might enhance their usual care due to the surplus of information collected with the EasyCare-TOS. However, these possible effects will comparably occur in the participants and professionals of the intervention group. Since we intend to pragmatically study the effects of the CareWell-primary care program in comparison to ‘care as usual’, these facts do not threaten our study as the focus will be on the additional value of our integrated care program in comparison to ‘usual care’, that is conducted following the EasyCare-TOS.

In combining the (cost-) effectiveness study with a thorough process evaluation, we will be able to draw conclusions not merely on the (cost-) effectiveness of the program, but, moreover, on the influence of the degree and process of implementation of the program on its efficacy. Moreover, we will be able to evaluate the feasibility of a nationwide implementation and structural financing of the program within the Dutch health care system.

## Abbreviations

ADL: Activities of daily living; EasyCare-TOS: EasyCare Two-step Older persons Screening instrument; GP: General practitioner; ICER: Incremental cost-effectiveness ratio; QALY: Quality-adjusted life years; ZWIP: Health and welfare information portal.

## Competing interests

The authors declare that they have no competing interests.

## Authors’ contributions

FR and AM drafted the manuscript. GvdW contributed to the construct of the economic evaluation. BvG contributed to the construct of the process evaluation. RA was involved in the methodological construct of the study. SZ, HS, TvA and RK designed the study and wrote the grant application. All authors are actively involved in the study and approved of the final draft of this manuscript.

## Pre-publication history

The pre-publication history for this paper can be accessed here:

http://www.biomedcentral.com/1471-2296/13/115/prepub

## Supplementary Material

Additional file 1 EasyCare-TOS step 2 questionnaire. Click here for file
